# Recurrent Rheumatic Carditis Presenting With Severe Biventricular Dysfunction Despite a Normally Functioning Mechanical Mitral Valve

**DOI:** 10.1016/j.jaccas.2025.106068

**Published:** 2025-11-20

**Authors:** José Victor da Nóbrega Borges, Samira Abdel Correia Leila

**Affiliations:** a Florida International University and Baptist Health South Florida, Miami, Florida, USA; b Hospital Israelita Albert Einstein, São Paulo, Brazil

**Keywords:** acute heart failure, mitral valve, rheumatic heart disease

## Abstract

**Background:**

Rheumatic heart disease (RHD) remains a major cause of cardiovascular morbidity in endemic regions. Recurrence of acute rheumatic fever (ARF) in adults, especially after valve surgery, is rare but clinically significant.

**Case Summary:**

A 44-year-old man with rheumatic mitral stenosis underwent mechanical mitral valve replacement. He presented 5 years later with dyspnea, orthopnea, and fatigue. Echocardiography showed new severe biventricular systolic dysfunction (left ventricular ejection fraction 20%) with preserved prosthetic function. Coronary computed tomography angiography excluded obstructive disease, and pulmonary embolism was ruled out. Elevated inflammatory markers raised suspicion for recurrent rheumatic carditis. He received anti-inflammatory therapy and guideline-directed medical therapy for heart failure, with progressive recovery.

**Discussion:**

Recurrent ARF should remain a diagnostic consideration in adults with prior RHD and new ventricular dysfunction, even after valve replacement.

**Take-Home Messages:**

Recurrent ARF may occur post-surgery and cause myocardial dysfunction. Echocardiography and multimodality imaging are essential for diagnosis and management.

## Central Illustration

**Figure:**
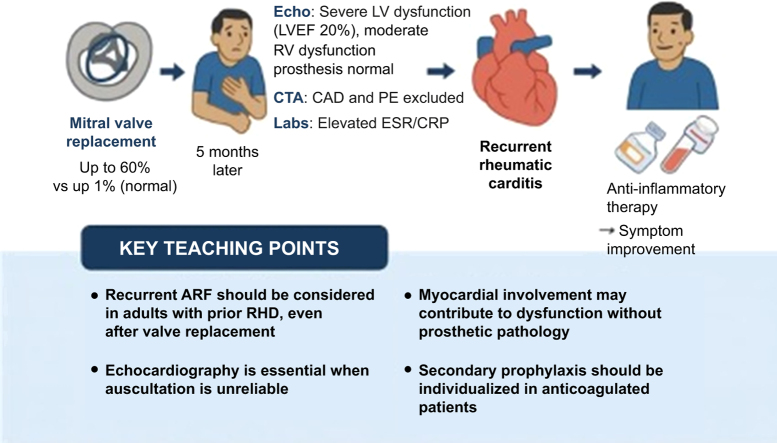

**Visual Summary:**
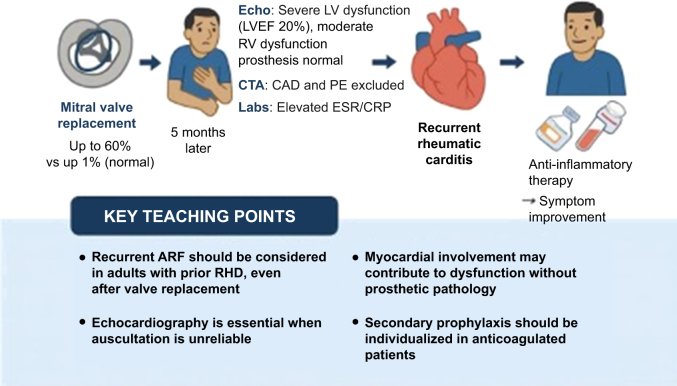
Clinical Course of a 44-Year-Old Man With Prior Rheumatic Mitral Stenosis Who Underwent Mechanical Mitral Valve Replacement and Later Developed Recurrent Rheumatic Carditis The patient presented 5 months postoperatively with new-onset dyspnea, orthopnea, and peripheral edema. Echocardiography revealed severe left ventricular (LV) systolic dysfunction (left ventricular ejection fraction [LVEF] 20%) and moderate right ventricular (RV) dysfunction, with normally functioning prosthesis. Coronary and pulmonary computed tomography angiography (CTA) excluded obstructive coronary artery disease (CAD) and pulmonary embolism (PE), and laboratory testing demonstrated elevated inflammatory markers. Recurrent rheumatic carditis was diagnosed and managed with anti-inflammatory therapy and guideline-directed medical therapy for heart failure, resulting in symptomatic improvement. Key teaching points emphasize the importance of considering recurrent acute rheumatic fever (ARF) in adults after valve replacement, the pivotal role of echocardiography, recognition of possible myocardial involvement, and the need for individualized secondary prophylaxis in patients receiving anticoagulant therapy. CRP = C-reactive protein; ESR = erythrocyte sedimentation rate; RHD = rheumatic heart disease.

Rheumatic heart disease (RHD) is a major cause of cardiovascular morbidity and mortality in low- and middle-income countries, with acute rheumatic fever (ARF) as its precursor.^1^^,^^2^ Whereas most cases manifest in childhood, recurrence in adulthood is well described, particularly in patients with prior RHD and incomplete secondary prophylaxis.^3^^,^^4^ In the postoperative setting, new heart failure warrants evaluation for prosthetic dysfunction, ischemia, pulmonary embolism, arrhythmia, and rarer causes such as recurrent rheumatic carditis. This case illustrates a rare presentation of recurrent ARF with severe biventricular dysfunction following mechanical mitral valve replacement, emphasizing diagnostic and management considerations in this context.

**Take-Home Messages**

• Recurrent ARF should be considered in adults with prior RHD and new ventricular dysfunction, even post–valve replacement.
• Echocardiography is pivotal when auscultation is unreliable.
• Myocardial involvement in ARF, though uncommon, may contribute to dysfunction without prosthetic pathology.
• Secondary prophylaxis should be individualized for patients receiving long-term anticoagulant therapy.

## Case Presentation

A 44-year-old man with severe rheumatic mitral stenosis underwent successful mechanical mitral valve replacement. Early postoperative echocardiography showed preserved left ventricular ejection fraction (LVEF) (60%), normal prosthetic function, and no other significant valvular disease.

The patient presented 5 months later with progressive dyspnea, orthopnea, and peripheral edema. Physical examination revealed elevated jugular venous pressure, bibasilar crackles, and peripheral edema. Cardiac auscultation detected a normal prosthetic S_1_ click without new murmurs.

Transthoracic echocardiography demonstrated severe global left ventricular hypokinesia (LVEF 20%), moderate right ventricular dysfunction, and normal mechanical mitral prosthesis function. Coronary computed tomography (CT) angiography showed no obstructive disease; CT pulmonary angiography excluded pulmonary embolism. Laboratory testing revealed elevated erythrocyte sedimentation rate and C-reactive protein. Blood cultures were negative.

Given the acute decline in ventricular function, absence of prosthetic dysfunction, and elevated inflammatory markers, recurrent rheumatic carditis was suspected. A comprehensive work-up for other etiologies of myocarditis, including viral serologies (coxsackievirus, adenovirus, parvovirus B19, cytomegalovirus, Epstein-Barr virus), autoimmune markers (antinuclear antibody, antineutrophil cytoplasmic antibody), and thyroid function, was unremarkable. Although antistreptolysin O titers were not obtained, the combination of elevated inflammatory markers, typical imaging findings on fluorodeoxyglucose positron emission tomography (PET), and exclusion of alternative etiologies supported the diagnosis of recurrent rheumatic carditis. The patient received anti-inflammatory therapy along with guideline-directed heart failure management, resulting in gradual symptomatic improvement.

## Discussion

RHD accounts for up to 15% of heart failure cases in endemic areas.^1^^,^^2^ Although ARF most often occurs in childhood, recurrence in adults is recognized, especially in those with prior RHD.^3^^,^^4^

Adult ARF can mimic postoperative complications such as prosthetic dysfunction, ischemia, pulmonary hypertension, tachyarrhythmia-induced cardiomyopathy, or viral myocarditis.^3^^,^^4^ The 2015 American Heart Association (AHA) revision of the Jones criteria emphasizes Doppler echocardiography, including detection of subclinical carditis, and provides risk-stratified thresholds for diagnosis.^5^ In patients with valve replacement, auscultatory findings are often unreliable, making imaging central to diagnosis.^5^^,^^6^

Rheumatic carditis typically causes ventricular dysfunction through valvulitis-related regurgitation rather than primary myocarditis.^7^ However, cardiac magnetic resonance and histopathology studies demonstrate that myocardial inflammation can occur in ARF and may contribute to dysfunction.^8^ In this case, severe biventricular dysfunction despite a normally functioning prosthesis raises the possibility of myocardial involvement.

**Figure 1:**
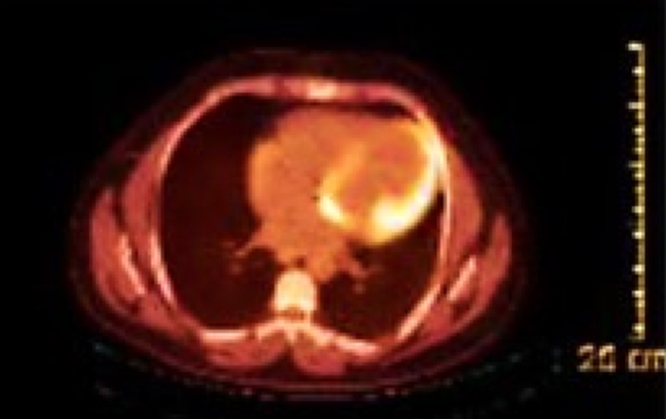
Positron Emission Tomography/Computed Tomography With Fluorodeoxyglucose Showing Increased Myocardial Uptake Consistent With Active Inflammatory Process (Maximum Standardized Uptake Value 6.6)

**Figure 2:**
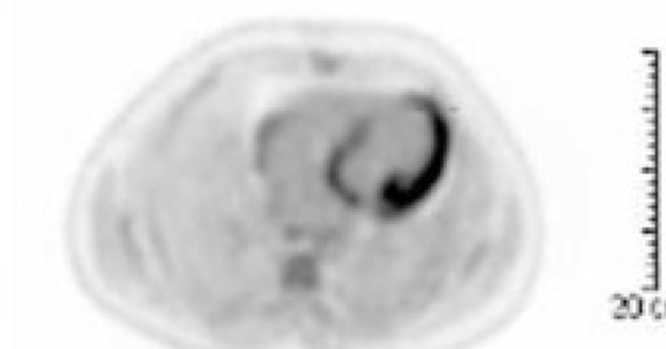
Fused Positron Emission Tomography/Computed Tomography Images Demonstrating Focal Fluorodeoxyglucose Myocardial Hypermetabolism (Maximum Standardized Uptake Value 6.6)

**Figure 3:**
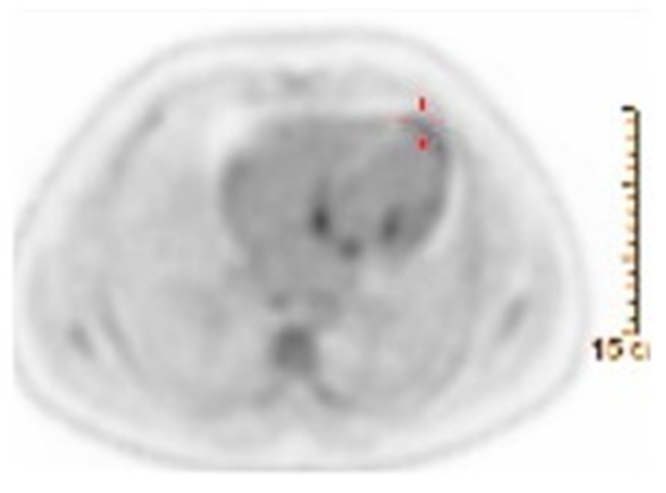
Repeat Positron Emission Tomography/Computed Tomography Demonstrating Partial Reduction of Myocardial Inflammatory Activity (Maximum Standardized Uptake Value 4.4) A new course of prednisone 80 mg/d was initiated for 3 weeks, followed by gradual tapering.

**Figure 4:**
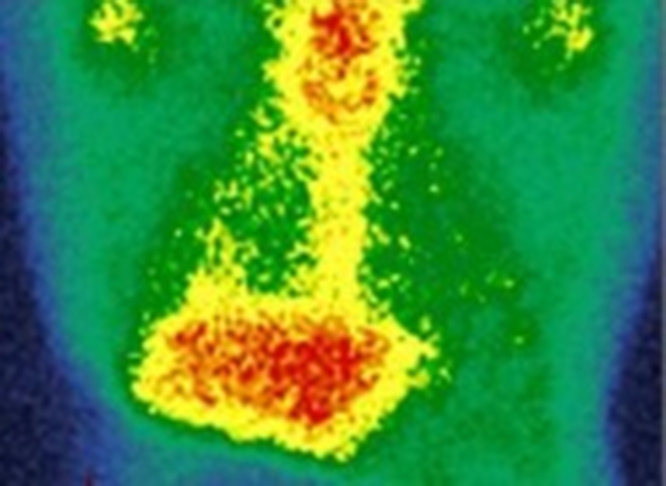
Follow-Up Gallium-67 Scintigraphy Demonstrating No Evidence of Active Cardiac Inflammatory Process

In this case, the diagnosis of recurrent rheumatic carditis was based on clinical presentation, inflammatory markers, and characteristic PET/CT findings in the context of previous rheumatic disease and exclusion of alternative causes of myocarditis (Figures 1, 2, 3, and 4). Although antistreptolysin O and anti-DNase B titers can support recent streptococcal infection, they are often negative in late recurrences or in patients previously exposed to antibiotics. Therefore, imaging and clinical context remain critical for diagnosis in adults.

Anti-inflammatory therapy, salicylates or nonsteroidal anti-inflammatory drugs (NSAIDs), remains first-line treatment, with corticosteroids reserved for patients with severe carditis or heart failure requiring rapid inflammation control.^9^ Trials show no superiority of corticosteroids over NSAIDs in preventing chronic valve disease.^9^ Heart failure should be managed according to guideline-directed therapy, with attention to prosthetic valve hemodynamics.^9^

Secondary prophylaxis is critical to prevent recurrence. The AHA recommends intramuscular benzathine penicillin G every 3 to 4 weeks or oral penicillin V twice daily when intramuscular administration is not feasible.^10^ In patients receiving anticoagulant therapy, oral regimens may reduce injection-site hematoma risk, though adherence must be ensured.^10^

## Conclusions

Recurrent ARF can occur in adults after valve replacement and should be included in the differential diagnosis of new ventricular dysfunction in patients with prior RHD. Multimodality imaging is crucial for diagnosis, and management should combine anti-inflammatory therapy, guideline-directed heart failure treatment, and tailored secondary prophylaxis.

## Funding Support and Author Disclosures

The authors have reported that they have no relationships relevant to the contents of this paper to disclose.
